# Energy utilization in fluctuating biological energy converters

**DOI:** 10.1063/1.4945792

**Published:** 2016-04-28

**Authors:** Abraham Szőke, Janos Hajdu

**Affiliations:** 1 Lawrence Livermore National Laboratory, Livermore, California 94551, USA; 2 Department of Cell and Molecular Biology, Uppsala University, Box 596, S-75124 Uppsala, Sweden

## Abstract

We have argued previously [Szoke *et al.*, FEBS Lett. **553**, 18–20 (2003); Curr. Chem. Biol. **1**, 53–57 (2007)] that energy utilization and evolution are emergent properties based on a small number of established laws of physics and chemistry. The relevant laws constitute a framework for biology on a level intermediate between quantum chemistry and cell biology. There are legitimate questions whether these concepts are valid at the mesoscopic level. Such systems fluctuate appreciably, so it is not clear what their efficiency is. Advances in fluctuation theorems allow the description of such systems on a molecular level. We attempt to clarify this topic and bridge the biochemical and physical descriptions of mesoscopic systems.

## I. INTRODUCTION

Life on Earth is organized around the utilization of high grade (“available”) energy from photons, chemicals, and chemical gradients. Biological catalysts are capable of controlling the flow of energy by an “almost” reversible exchange of high-grade energy between themselves and their reactants, thereby changing the path of chemical reactions and influencing their rate.^1,2^ The primary energy is seldom used directly. Energy is transferred in small quantities because higher energy densities can be harmful to the system. Energy converters working in cells enable the reduction of entropy generated in reactions, in order to maintain structures, the synthesis and degradation of chemicals, as well as the duplication of templates.

In this paper, we focus on tracking the flow of available energy in small systems that are surrounded by an environment with a well-defined temperature. This environment constitutes a thermal bath, which fluctuates on the molecular and mesoscopic level. Brownian motion is a direct consequence of such fluctuations. The reacting system is so small that it cannot influence the surrounding thermal bath by any appreciable amount. The average ratio of the forward to backward rates and the macroscopic reversibility of a process are, therefore, determined by the net ΔG.

In 1976, Cooper reported calculation of the mean square fluctuation in enthalpy of a single protein molecule of 25 000 kDa mass^3^ and found that the RMS fluctuations were approximately 38 kcal per mole, or about 66 *k_B_T*, if all molecules were to fluctuate in synchrony. Most chemical reactions in biology involve energy changes less than a tenth of this amount. Cooper's calculations reveal the importance of structure in creating the mechanisms of utilising high-grade energy in biology. In such systems, structure is organizing random thermal motions into defined landscapes that allow the mobilization of free energy. Experimental demonstration of fluctuation in the activity of individual lactate dehydrogenase (LDH) molecules showed up to 5-fold changes in the activity of single LDH molecules over time.^4^ In most systems, fluctuations within a single molecule are uncorrelated with similar fluctuations in other molecules. In a population of many molecules, such fluctuations will cancel to give a sharp, essentially non-fluctuating, measurement of the thermodynamic parameters.^3^

The related, important question we will concentrate on is how to make sense of the role of ΔG in view of the fluctuations that are inevitable not only in the molecules studied but also even in its environment that is a thermal bath. Recent advances in “fluctuation theorems” allow the discussion of such systems on a molecular level. We apply this framework to a set of protein machines that perform work.

Primary available energy in living systems is either from light, from chemicals, or from chemical gradients. The primary energy is seldom used directly in life. Most of the chemical-metabolic apparatus functions by converting one kind of high-grade energy into others, inevitably generating some heat (entropy) in an irreversible process.

We called such systems energy converters or, somewhat poetically, the “engines” of life. In this paper, we will apply our framework to some of the engines of life. First, to set the stage, the concept of emergence is reviewed and the mesoscopic–molecular level of description is defined.^2^

## II. EMERGENCE

It is accepted wisdom that properties of living systems are based on well-established laws of physics and chemistry.^5,6^ Physical laws provide the necessary conditions for understanding biological phenomena, but biological systems—like all other complex systems—should be described and understood on several distinct levels. Within each level, a new set of rules has to be established, appropriate to that level. These rules and their consequences have been called emergent behavior. The emergent rules of biology must not contradict the laws of physics and chemistry, but they have to be established in addition to them.^7^ Some of these ideas have been present in biological thinking for many years, witness Monod's book, Chance and Necessity.^8^ Papers of Laughlin and Pines,^7^ Woese,^9^ and Noller^10^ as well as the classic works of Dawkins^11,12^ and Eigen^13,14^ illuminate the subject. Our main interest here will be the utilization of available energy by small living systems. For that purpose, three layers, or levels of description suffice: macroscopic, mesoscopic, and microscopic.

### A. Macroscopic and microscopic descriptions

At the macroscopic level, thermodynamics is valid and fluctuations are negligible. In particular, the concepts of free energy, of reversible and irreversible processes are well defined. At the microscopic level, both Hamilton's equations of motion^15^ and Schrodinger's equation are time reversible. The concept of irreversibility in statistical mechanics is far from simple to introduce, and it is applied mostly to macroscopic systems. In Secs. III–VII, we attempt to show that some macroscopic concepts have meaning on the mesoscopic level, enabling us to describe energy utilization in a physically, chemically, and biologically meaningful way.

### B. Mesoscopic description

The mesoscopic size range includes enzymes, RNA and DNA molecules, membranes, ribosomes, cell organelles, molecular machines, etc. They act inside living cells and have well defined functions. Textbooks of molecular biology^5^ and biochemistry^6^ discuss life on this level. Nevertheless, our approach is different; it centers on energy utilization and, in particular, on the meticulous distinction between high-grade energy and heat (e.g., Refs. 1 and 2).

A central question in such a mesoscopic description is the applicability of macroscopic concepts of thermodynamic potentials, reversibility, and the generation of irreversible entropy. Small systems, when exposed to an environment in thermal motion, fluctuate appreciably. This is especially true when processes are very fast and when they occur under non-equilibrium conditions. In fact, intuitively, one could doubt that such concepts are valid at all. Note that on the microscopic level of quantum chemistry, the equations of motion are reversible and the forces are always gradients of potentials that we enumerated as high grade energy.^1,2^ It is also true that free energy—as well as temperature, pressure, etc., that follow from it—are usually introduced “externally” in quantum chemistry calculations and in molecular dynamics simulations.^16^ We will now argue that fluctuation theorems come to our rescue.

## III. FLUCTUATION THEOREMS, I

Statistical mechanics deals with large, ergodic systems.^17^ Many fundamental theorems of thermodynamics (e.g., the first and the second law) are valid for equilibrium system and for processes where the system stays close to equilibrium. In recent years, there has been a large and increasing interest in “fluctuation theorems” that are able to deal with small systems and also with processes far from equilibrium.

Ergodicity means that the time average and the space average are the same. Here, we are interested in a single unit of a system. Such a system fluctuates because it is embedded in a thermal bath. We deal with time averages here where ΔG determines the time average of the fluctuations. This common thread illuminates an underlying simplicity.

In this section, we present fluctuation theorems that have a somewhat restricted validity. They analyze the “bioenergetics” of molecular processes, where a small system is embedded in a larger system that is close to thermal equilibrium. It is called a thermal bath. The process may be a catalyzed reaction, the unfolding of a protein, the synthesis of adenosine triphosphate (ATP), or the transport of vesicles by kinesin; all of them embedded in a living cell. The additional important assumption of the theorems presented below is that the subsystem starts in a thermodynamic, equilibrated state; it then undergoes a process and ends up in another thermodynamic state. During the process, the small system can be far from any thermodynamic state: as an example, its internal degrees of freedom can be in complicated states of motion. For example, during catalyzed reactions, the catalyst and the reactants may have very complex interactions.

There are two main strands of research on fluctuation theorems. One is associated with Gallavotti and Cohen^18^ and with Evans and Searles.^19^ The other strand is associated with Jarzynski^20^ and Crooks.^21^ Extensions to processes where quantum mechanics is important have been discussed by Chernyak and Mukamel.^22^ Recently the theorem has been verified by experiment^23^ and has been used to determine the free energy differences of molecular processes; reviews of earlier work can be found in Refs. 23 and 24.

Let us suppose an external force is applied during a process that connects one thermodynamic equilibrium state *A* with another equilibrium state *B*. Denoted by *W* is the work carried out by the external force. If the system is small, *W* fluctuates from repetition to repetition of the process. When the process is carried out in the reverse direction, the work is denoted by –*W*. It also fluctuates between repetitions. Jarzynski's theorem^20^ states that, at temperature T,

$$\text{exp}[-\Delta G/k_{B}T]=\langle \;\text{exp}[-W/k_{B}T]\rangle ,$$ (1)

where the angular brackets ⟨ ⟩ denote averages over many repetitions of the experiment and *k_B_* is Boltzmann's constant.^25^ Here, ΔG is the change of the Gibbs free energy in the forward process. Note that as both the initial and the final states are in thermal equilibrium with their surroundings, this difference is well defined. The equation states that the exponentially weighted average of the work expended in the process depends only on the free energies of the initial and final states, although it fluctuates from instance to instance. Unfortunately, the equation as presented here is not very useful for experiments because the rare instances where *W* is very small will contribute exponentially to the average.

Another version of the theorem, by Crooks,^21^ discusses the relation between the work, *W* and the probabilities of a forward transition, P(A→B;W) to the backward transition, P(B→A;W) at a given *W*,

$$\frac{P(A\to B;W)}{P(B\to A;W)}=\text{exp}(\frac{W-\Delta G}{k_{B}T}).$$ (2)

If the external work is kept constant, the ratio of the forward to backward probabilities can be measured. We also note that the quantity W−ΔG is the heat generated in the transition if the temperature of the environment is constant. This equation will be very useful to us below.

The fluctuation theorem has an alternative interpretation: It shows that the average behavior of catalyzed chemical reactions obeys the rules of macroscopic thermodynamics, at least within the limits set by fluctuations and as long as classical concepts are valid. In particular, the theorem states that thermodynamics is valid for single enzymatic processes, as long as we do not try to calculate reaction rates *ab initio*, or enquire about detailed mechanisms of the partition of the energy between high grade and low grade energy, or about the mechanism of irreversible entropy generation. Note that at the level of quantum chemistry the equations of motion are time-reversible and all forces are gradients of potentials. The concept of heat generation can be tackled only by introducing chaotic trajectories. Note also that the description of chemical reactions by transition state theory is fully compatible with the fluctuation theorem. All our statements in the Introductory section of this paper are also compatible with the fluctuation theorem.

The previous statements should not come as a complete surprise. After all, the concepts of chemical equilibrium were established more than a hundred years ago on the basis of classical statistical mechanics and they are fully compatible with them. In other words, if we look at the *average* behavior of individual molecules, they are described correctly in textbooks of physical chemistry. Moreover, their states fluctuate and the magnitudes of the fluctuations are also correctly described by the “classic” fluctuation-dissipation theorem.^17^

An important corollary of the fluctuation theorem is that the average ratio of the forward to backward rates and the macroscopic reversibility of a process are determined only by the net ΔG. This, of course, agrees with classical thermodynamics. It should come, therefore, as no surprise that proton pumps, molecular motors or $F_{0}-F_{1}\;\text{ATPase}$ sometimes step “backwards.” In fact, as elaborated below, a measurement of the backward stepping probability can give a quantitative estimate of the actual reversibility of any of these processes.

We present now some additional analysis that will be useful for interpreting experiments. The simplest one starts from Eq. (2). Suppose that the experiment is repeated many times with the work *W* kept fixed.^26^ In that case, we can average the probabilities separately, to read

$$\langle P(A\to B)\rangle =\text{exp}(\frac{W-\Delta G}{k_{B}T})\langle P(B\to A)\rangle ,$$ (3)

and finally,

$$\langle R\rangle =\text{exp}(\frac{W-\Delta G}{k_{B}T}).$$ (4)

The equation shows that the experiment will sometimes go from the starting state to the end state and sometimes the other way around. The experiment provides then a statistical sample of ⟨R⟩ and, by Eq. (4), an estimate of ΔG. Standard statistical analysis shows that the estimate converges much faster and has less bias than the average of $\left\langle \;\text{exp}[-W/k_{B}T]\right\rangle$ in Eq. (1), where the initial and the end states are fixed and the work *W* is left fluctuating. In the experiments analyzed below, the force acting on the molecule and the length of a “step” are indeed kept constant, giving W=Fd=const. Section VII of the paper proposes a new theoretical method to obtain ΔG based on this observation and presents an analysis of its advantages.

## IV. FLUCTUATION THEOREMS, II

There has been a lot of recent progress on answering the question what happens in processes that are *always* far from equilibrium. A very condensed, but clear summary is given by Van Den Broeck and Esposito.^27^ A more detailed review is by Seifert.^28^

The short answer is that essentially all the thermodynamic concepts are valid even in such cases. In the following, we present some details.

We enquire about a “process” in a living cell, where a small part of the cell participates in some chemical-physical reactions (for example, a glucose+ATP→glucose-6-phosphate+ADP (adenosine diphosphate) reaction). We assume that there is a statistical ensemble of states at the start of the reaction. We *do not* assume that either before, during, or after the process any of the reactants or the products are in thermal equilibrium at any time. We also assume that the cell is large enough that it has a well defined temperature and that it is not affected by the reaction. In other words, it provides a “thermal bath.”

At the beginning of the process, the ensemble of the reactants is in a distribution of states *P*(*m*), where *m* labels the individual “micro” states, that we assume we know. For example, *ATP* and glucose are in their ground electronic states and vibrational states and in thermal equilibrium in their rotations and translations. The most important concept introduced by “statistical thermodynamics” is the entropy

$$S=-k_{B}\sum_{m}P(m)lnP(m).$$ (5)

Here, *k_B_* is the Boltzmann constant. This formalism has newly been shown to apply to systems always far from equilibrium.^27^ Similarly, the energy and the number of particles in the ensemble are defined as

$$E=\sum_{m}\epsilon_{m}P(m),$$ (6)

$$N=\sum_{m}n_{m}P(m).$$ (7)

Here, *ϵ_m_* and *n_m_* denote the energy and the occupation number of the micro state *m*, respectively.

The beauty of this formulation is that the first and second laws of thermodynamics maintain their meanings even for small ergodic systems. The first law (conservation of energy) is

$$\dot{E}=\dot{Q}+{\dot{W}}_{chem}+\dot{W}.$$ (8)

The symbols need some explanation. The chemical work rate is ${\dot{W}}_{chem}=\mu \dot{N}$, where *μ* is the chemical potential and $\dot{N}$ is the time derivative of the number in the ensemble. The ordinary work rate term $\dot{W}$ appears in the equation if there is an external potential that varies in time. It can work on the system or work by the system.

The change in entropy can be divided into an external part and an internal part

$$\dot{S}={\dot{S}}_{e}+{\dot{S}}_{i}.$$ (9)

The external part is connected to the heat coming from the environment

$${\dot{S}}_{e}=\dot{Q}/T.$$ (10)

The change in the internal part of the entropy is always non-negative

$${\dot{S}}_{i}\geq 0.$$ (11)

It is zero for a reversible process. That is when the maximum work can be extracted from an adiabatic process. Then $\dot{S}=\dot{Q}/T$, just as in the second law of classical thermodynamics.

If we concentrate on individual reactions on a reaction path, also called a trajectory, all these relations are still valid.

When a definite trajectory is considered, a “time reversed” trajectory can be defined. It is obtained if all the positions of the particles are kept at the end of the forward trajectory, but the momenta are reversed, the external forces are similarly reversed, and their dependence on time is also reversed. This is the way the fluctuation theorems, discussed in Sec. III, were obtained.

An equation similar to (4) for the change in the internal entropy on a trajectory was obtained by Seifert^28^

$$\frac{P(\Delta s)}{\tilde{P}(-\Delta s)}=\text{exp}(\frac{\Delta s}{k_{B}}).$$ (12)

Here, P(Δs) is the probability for observing a change in entropy, (Δs) on the forward trajectory and $\tilde{P}(-\Delta s)$ is the probability of observing a change in entropy (−Δs) on the reversed trajectory (if it starts immediately at the end of the forward trajectory). This shows that it is exponentially more likely to observe an increase in entropy than its decrease. We also note the similarity to Eq. (4) that shows that for a unidirectional trajectory, one needs to lose free energy. Although the derivation of Eq. (4) depended on the assumption of thermal equilibrium in the initial state, Eq. (12) shows that the fluctuation theorems have an even wider validity.

Another important question is how much of the extractable free energy is lost if the non-equilibrium state at the end of the process relaxes into an equilibrium state irreversibly, i.e., without doing any work. In other words, how much of the possible work is lost in this process. The difference in entropy between the unrelaxed state and the relaxed state is given by the relative entropy

$$S_{rel}=-k_{B}\sum_{m}P(m)ln(\frac{P(m)}{P_{eq}(m)}).$$ (13)

Here, $P_{eq}(m)$ is the equilibrium distribution to which it relaxes. Some of this can be extracted as external work; it was calculated by Sivak and Crooks^29^ for states that are not too far from equilibrium. It essentially states that half of this heat *TS_rel_* can be extracted as work and the other half is always lost.

## V. SOME CLARIFICATIONS

We end our general discussion with two comments.

As an example, the initial state of the reaction—glucose+ATP→glucose-6-phosphate +ADP—can be considered an equilibrium state in the sense that the reactants are in (translational and rotational) thermal equilibrium with the solvent. After the first step of the reaction, when *ATP* dissociates to *ADP* + *Pi*, the free energy released must go into some internal stress (i.e., potential energy) of the molecule. There are two parts to this statement: first, the exoergic step must be the first one, because otherwise the enzyme will not work. Second, the free energy in the reacting system must decrease in time, otherwise the reaction would not proceed predictably. It is this intermediate state where our long-winded argument is important. In much simpler reactions, e.g., when only a proton is transferred, there is no intermediate state.

We promised that we will try to translate the lexicon used by the practitioners of “bioenergetics” to the nomenclature of the rest of science. Their use of “energy conservation” is the same as our use of “utilization of free energy.” There are several problems with their usage. First, energy is always conserved in nature. The first law of thermodynamics is the conservation of energy, and it is described in Eq. (8). The subtle point is that part of the energy is converted into heat that goes into the environment and thus it is not available to do useful work anymore. Bioenergetics is really about the same distinction, and it is unfortunate that they use such a vague definition.

## VI. ENERGY CONVERTERS: THE ENGINES OF LIFE

The primary energy is seldom used directly in life. Most of the chemical–metabolic apparatus functions by converting one kind of high-grade energy into others, usually generating some heat in the process. We labeled such systems the engines of life. In biology, energy converters are found that convert each type of high grade energy—namely, photon energy, chemical energy, electrical energy, and mechanical energy—into each other type. Here, only a very partial list of the kinds of energy converters and an even sparser set of examples are given. Finally, the linear motor, kinesin is discussed in some detail.

### A. Utilization of chemical energy

From an energy perspective, almost all enzymes—in fact most metabolic processes—function by interchanging (transferring, converting) chemical energy among different molecules. In the simplest case, enzymes select a reaction with a small ΔG, among all possible ones. We called them reaction selectors.^1^ The free energy “conserved” this way is available for the organism for later use. In coupled reactions, a strongly exoergic reaction drives a less endoergic reaction uphill. The fundamental mechanism of coupled reactions is that the driving reaction excites the catalyst by a free energy conserving process; then the catalyst transfers its excess free energy into the uphill reaction.

A very simple example is the first step of glycolysis. The enzyme hexokinase enables a phosphate group from ATP to attach to glucose instead of being hydrolyzed. The overall reaction of—glucose+ATP→glucose-6-phosphate+ADP—has negative ΔG. (It loses free energy, i.e., it generates heat.) Nevertheless, much of the free energy of the phosphate bond gets “invested” into the newly formed glucose-6-phosphate. The role of the enzyme is to lower the barrier for the reaction that conserves free energy, relative to the barrier for hydrolysis.^30,31^ This way the reaction is redirected into the desired direction.

An important remark about reaction selectors and coupled reactions is that it is absolutely essential that the free energy of the “driving” reaction is transferred directly into the product or the catalyst, respectively: i.e., it stays as a high grade (available) energy and only the difference between the free energies is converted into heat. This remark puts a stringent restriction on the possible mechanisms of coupled reactions. Unfortunately, while the above statement is not contradicted in most of the relevant literature, it does not appear explicitly in many modern textbooks or review papers. A simple way to understand the energetics of coupled reactions is by referring to the formula

$$\Delta G=\Delta G_{0}+k_{B}Tln([D][E])/([B][C]).$$ (14)

The equation describes the dependence of ΔG on the concentrations of the reactants and products in a reaction, B+C⇄D+E. As usual, the letter *B* denotes a chemical species and [B] denotes its concentration (or actually its fugacity, to be pedantic). The standard free energy of the reaction is denoted by $\Delta G_{0}$. It occurs when the concentration of the reactants and the products are equal. Chemical equilibrium is established when ΔG=0. An enzyme that speeds up the B+C⇄D+E reaction and, possibly, changes the local G of the reactants can be perceived as an engine that establishes partial equilibrium in the cell (or within the enzyme itself).

### B. Conversion of photon energy into electrical energy

The most important energy input of life on earth is solar radiation. All absorbed photons are converted into electrical and/or chemical energy. The energetics of the first steps of photosynthesis are well described in books on bioenergetics. We will review them briefly here. Both the reaction center (a single one in simple bacteria) as well as bacteriorhodopsin (in archaea) convert the free energy of the absorbed photon into an electro-chemical potential across a membrane that embeds the molecule. From the point of view of available energy, there is no difference between an electric potential and a pH gradient with the proper conversion factors.^32^ In this light, both the reaction center and bacteriorhodopsin can be considered to be battery chargers. We will call the membrane “charged,” expressing our physics as opposed to chemistry perspective.

A bacteriochlorophyll molecule in the *Rhodobacter sphaeroides* reaction center efficiently absorbs a photon^33^ and gets electronically excited. The excited electron then jumps to a neighboring bacteriochlorophyll molecule and then to a bacteriopheophytin molecule.^34^ The original molecule (one of the “special pair”) is now ionized, and the bacteriopheophytin is now a negative ion. There are two important considerations: First, the resulting free energy of the ion pair has to be less than the original electronic excitation energy of the bacteriochlorophyll molecule. Second, as the bacteriopheophytin is positioned deeper in the charged membrane, part of the electron free energy is converted into reversible work against the electric field in the membrane. The next step in the electron transfer chain brings the electron even deeper into the membrane to a molecule of ubiquinone. Finally, ubiquinone is neutralized by a proton translocated from the other side of the membrane, again doing reversible work on the electric field.^35^ Note that, when the pheophytin molecule is neutralized, the battery is already charged so some of the photon energy has been converted into an electro-chemical potential at this point. At a later stage the pheophytin molecule is used as chemical energy, but that is another story.

Bacteriorhodopsin presents an even cleaner case, although its energetics seems not to be clearly described in the literature. A photon is absorbed in the retinal molecule that is bound to the protein. Within a few picoseconds, a (negative) ion moves over to the other side of the retinal and the retinal itself changes its structure: it undergoes a *cis-trans* transformation.^36^ It is possible that the excited electron itself jumps onto a neighboring molecule, producing an ion pair, just as in the reaction center. The ion pair formation can only be inferred through spectroscopy, but the *cis-trans* transformation is clearly seen in time dependent crystallography.^37^ At this point, both the reversible work of the charge separation against the electric field in the membrane and the change in the chemical energy of the rhodopsin have to be counted as high grade energy. At the later stages of the bacteriorhodopsin cycle, the charge separation increases and, finally, one electron charge is completely transported across the membrane, against the electric potential. Note that this transport is very slow on the time scale of molecular relaxation; therefore, the initial charge separation *has* to have a higher free energy than the membrane potential. Finally, the retinal reverts to its cis configuration. At this last stage, all the energy investment in the *cis-trans* transformation is irreversibly lost. (It is converted into heat.) We can surmise that the important role of the *cis-trans* isomerization is to prevent the recombination of the initial charge separation, as has been suggested for the related halorhodopsin.^38^

### C. Conversion of electrical energy into mechanical energy

Electrical energy in cells is often converted into mechanical energy. Prime examples are the flagellar motor and the $F_{0}\;\text{ATPase}$. Note that $F_{0}\;\text{ATPase}$ is always coupled to $F_{1}\;\text{ATPase}$ that converts the mechanical energy generated into chemical energy by generating ATP. We find it remarkable that the whole transformation of electrical to chemical energy in the coupled $F_{0}-F_{1}\;\text{ATPase}$ is reversible. The overall efficiency of this process is claimed to be 90%,^39^ which intuitively seems high.

Both the flagellar motor and the $F_{0}\;\text{ATPase}$ are built similarly and function by a very similar mechanism. They span a lipid membrane and they are rotary motors. Their central part is a multi-segmented rotor; it is surrounded by a multi-segmented stator that is anchored in the membrane. The mechanism is based on static electricity, not on electromagnetic effects like electric motors built by human engineers. The individual driving members are linear, although the result is a rotary motion. The driving high grade energy is the electrochemical potential across the membrane.^40^ That potential is the combination of the electrostatic energy of the membrane as a capacitor and the concentration gradient across it.^2^ When protons (and/or electrons) cross the membrane, a current flows and the motor extracts the high grade energy stored in the electrochemical potential.

The coupling between protonation and rotation in flagellar motors is subtly controlled. For instance, a simple Asp to Glu mutation in Typhimurium MotB impairs motility in a manner that is poorly understood.^41^ A critical component of the operation of the motor is that at either end of the stroke the protonation and deprotonation of the functional residue is (almost) reversible. One prediction is that there is a maximum potential that the reversed motor can produce and the same potential is the minimum at which the motor starts working. Another prediction is that if the potential is higher than the minimum, the excess potential is dissipated into heat. That would yield the required irreversibility for reliable operation of the motor. If the torque of the rotor is larger than the electric force during the charged stage of the cycle, the tongue moves against the electric force and charges the membrane. The mechanism outlined above is speculative, but it agrees with data on both the flagellar motor and the $F_{0}\;\text{ATPase}$ in the literature.^42–47^

## VII. CONVERSION OF CHEMICAL ENERGY INTO MECHANICAL ENERGY; LINEAR MOTORS; KINESIN

### A. Linear motors

Conversion of chemical energy into mechanical energy and, eventually, into motion occurs widely in living cells. Let us mention linear motor proteins, G-proteins as well as the elongation factors EF-Tu and EF-G in the ribosome. Kinesin will be our paradigm, but many of the considerations below are applicable to all other linear motors, as well as to rotary motors and even to ion pumps. In some sense, the operation of all known linear motors is similar. It is based on the conversion of the energy of a chemical bond into internal strain of the molecule.^48^ In a somewhat simplistic way, we can imagine that when the phosphate bond is broken it strains (pushes apart, or twists) the surrounding molecular structure. The picture is not completely unrealistic; we note that the atomic potentials are very short range—atoms have to move only an inter-atomic distance, or so, in order to release the bond energy. The picture is similar to our biceps lifting our forearms: by exerting a large force and moving a small distance we are able to lift a smaller weight over a large distance. Newton taught us that the product of the force by the distance, i.e., the work, is unchanged.

### B. Kinesin

Kinesin is a two-handed motor that steps along microtubules. It is able to move loads against the viscous force of the medium or against an applied external force. The mechanical energy produced by the motor is eventually dissipated by viscous friction in the medium. The motor is fueled by the ubiquitous $\text{ATP}+H_{2}O\to \text{ADP}+P_{i}$ reaction. There are several essential steps in the operation of the motor. First, ATP and $H_{2}O$ have to bind. Second, when ATP hydrolyzes, the free energy released has to be converted “instantly” into strain energy and into chemical energy of the molecule (with respect to the state of the molecule when it was attached to the microtubule). In our language, the final state of the hydrolysis is $\text{ADP}+P_{i}$ + a strained molecule. (The strained molecule is in a higher free energy state than the unstrained one.) As the strain energy of the molecule is also high-grade energy, only the difference between the total energies of the reactants, $\text{ATP}+H_{2}O$ and the products: – $\text{ADP}+P_{i}$ + a strained molecule are converted into heat. Third, the products, ATP and $P_{i}$, have to be released.

In recent years, many detailed studies of kinesin have been made. The internal structure of kinesin has been elucidated by Fletterick and collaborators; see, e.g., Ref. 49 and references therein. The classic kinetics was reviewed in Ref. 50. Optical tweezers were applied by Block and collaborators to get the detailed information on the step size, efficiency, and the number of steps a single molecule is capable of taking before detachment (called processivity); see Ref. 51 and references therein. It was established that the step size is ≈ 8 nm, equal to the repeat distance of the microtubule. Also, each step is fueled by the dissociation of a single ATP molecule. The steps themselves are faster then 30μs, and intermediate states (if they exist) were not found in the measurements. Also, the two-handed (or two-headed) kinesin walks hand-over-hand: the lagging hand detaches from the microtubule and overtakes the leading hand alternately on either side.^51,52^

Some studies using optical tweezers with a retarding force are particularly revealing.^53,54^ We refer also to three recent review articles.^55–57^ Backward, as well as forward steps were observed; in fact, under some circumstances, there were a series of backward steps. (The backstepping was processive.) Both the forward and the backward steps were the same size, ≈ 8 nm indeed. The ratio of the number of backward steps to the forward steps could be fitted by the formula $\left\langle R\rangle =\text{exp}(-\Delta G/k_{B}T\right)$, with ΔG depending on the load as $\Delta G=\Delta G_{R=1}-Fd$, where *F* is the applied force and d≈ 8 nm is the step length. It was found that ⟨R⟩ is *independent* of ATP concentration, in the range of 10μM–1 mM. When the motor “stalls” under the force *F_stall_*, the forward to backward probabilities are equal, ⟨R⟩=1. This happens at $F_{stall}=7\text{pN}$. The stalling force gives a $\Delta G_{R=1}=Fd\approx$ 30 kJ/mole. The standard free energy of the $\text{ATP}+H_{2}O\to \text{ADP}+P_{i}$ reaction is $\Delta G_{0}\approx$ 30 kJ/mole, but under typical cellular conditions, it is ΔG≈ 50 kJ/mole (see, e.g., Ref. 6, p. 376), and it depends on the concentration of ATP. Lau and co-workers have performed a theoretical analysis of kinesin using fluctuation theorems and arrived at similar energies as we do.^58^

### C. Interpretation

There are many excellent articles on the detailed mechanism of kinesin stepping, e.g., Refs. 59 and 60. We also refer to a recent review on the interpretation of the fluctuation theorem by Jarzynski.^24^ Here, we try to answer a more modest question: can the fluctuation theorem teach us something about kinesin?

We start by asking if the fluctuation theorem is applicable to our problem. Kinesin has long pauses between steps and every (forward) step is like any other one, so we can assume safely that during pauses kinesin has a well defined free energy, *G*_0_. We now take it from experiment that one molecule of ATP is reduced per step. Therefore, from Eqs. (3) and (4), we get that at a forward to backward stepping ratio of unity (stall) the measured $F_{stall}d=\Delta G_{R=1}$, independent of the detailed mechanism. In particular, even if the backward stepping mechanism is different from the mechanism of the forward step, as long as the molecule stays bound to the microtubule, the relation has to hold.

The measured value of $F_{stall}d\approx 60\text{pNnm}\approx 34\text{kJ}/\text{mole}$ is independent of ATP concentration. It is significantly lower than the available free energy of the $\text{ATP}+H_{2}O\to \text{ADP}+P_{i}$ reaction under conditions in the cell, the latter being ≈50kJ/mole. We conclude that this value is characteristic of the “power step” and there is a large irreversible component to kinesin stepping.

A more puzzling fact is that the actual rate of backward steps is independent of the applied force, when the retarding force it greater than the stalling force of ≈7pN. A possible interpretation is that a backward step converts the free energy of the driving reaction into heat, i.e., the free energy is completely wasted. This is somewhat surprising, because the $F_{0}-F_{1}\;\text{ATPase}$ is almost completely ( 90%) reversible and the Na/K pump are at least partly reversible. The Na/K pump can synthesize ATP in the endoergic reaction, $\text{ATP}+H_{2}O\leftarrow \text{ADP}+P_{i}$ and the $F_{0}-F_{1}\;\text{ATPase}$ can charge the membrane with good efficiency. On the contrary, the chemical energy of ATP is eventually converted into heat in the living cell. So it is not completely surprising that there is no clear experimental evidence that kinesin can generate ATP. In fact, recent work by Block and co-workers^56^ discusses futile cycles and backstepping as hydrolysis of ATP without any work being done.

The fact that $\Delta G_{R=1}\approx \Delta G_{0}$, the standard free energy of the reaction, implies that somehow the reactants and the products have equal concentrations at the time of dissociation. An attractive interpretation of this fact is that the overall reaction of $\text{ATP}+H_{2}O\to \text{ADP}+P_{i}$ is close to equilibrium at the stalling force *when the reactants and the products are still bound inside the cavity in the kinesin.* In the normal, forward stepping, the irreversibility (heat or entropy generation) must come from the steps of binding and unbinding the reactants and products of ATP hydrolysis.

If this interpretation is correct, it would also imply that the curve of lnR vs. *F* is a straight line and it does not have a curvature around *F_stall_*; this also seems to be born out by experiments.^53,54^ The experiments also imply that the retarding force “couples” strongly into the driving reaction, meaning that the external force pushes the reactants directly. A simple mechanical analogy is a nutcracker with an actively expanding nut.

The overall efficiency of the kinesin motor is the ratio of ≈30 kJ/mole to ≈50 kJ/mole, that is, ≈60%, but our argument indicates that the irreversibility comes from the “other” steps of the overall kinesin cycle, the binding and unbinding of the reactants and products. The mechanism and the kinetics of kinesin stepping are not explained in detail by the high efficiency of energy conversion, although some statements are clearly correct. It is essential that ATP binds to the hind arm that molecular strain is “instantaneous” after ATP hydrolysis. (In enzymology jargon, ATP hydrolysis is tightly coupled to molecular strain.) An attractive model is that ATP binding to the hind arm depends on the strain in the molecule, that in the absence of ATP or ADP the two arms bind tightly to the microtubule and that ATP hydrolysis releases the hind arm and that the molecular strain moves the hind arm forward to a position where the next repeat of the microtubule binds it strongly. In this statement, there is no denial of stochastic fluctuations in the position of the free arm of kinesin. Nevertheless, one has to be painfully aware of Feynman's proof that a stochastic ratchet is never a perpetuum mobile.^61^ It has been observed that the application of a retarding force slows down the stepping. This has been explained quantitatively by an increase in the barrier of the transition state for binding ATP—caused, possibly, by the strain on the hind arm by the retarding force.^50^ A recent experiment by Yildiz and Vale^62^ gives support to our discussion.
